# The Association between Exposure to Residential Indoor Volatile Organic Compounds and Measures of Central Arterial Stiffness in Healthy Middle-Aged Men and Women

**DOI:** 10.3390/ijerph19020981

**Published:** 2022-01-16

**Authors:** Suzanne E. Gilbey, Christopher M. Reid, Rachel R. Huxley, Mario J. Soares, Yun Zhao, Krassi B. Rumchev

**Affiliations:** 1 School of Population Health, Curtin University, Perth 6148, Australia; christopher.reid@curtin.edu.au (C.M.R.); m.soares@curtin.edu.au (M.J.S.); Y.Zhao@exchange.curtin.edu.au (Y.Z.); K.Rumchev@exchange.curtin.edu.au (K.B.R.); 2 School of Public Health and Preventative Medicine, Monash University, Melbourne 3800, Australia; 3 Faculty of Health, Deakin University, 221 Burwood Highway, Burwood 3125, Australia; r.huxley@deakin.edu.au

**Keywords:** indoor air quality, household air pollution, cardiovascular risk, VOC, sex differences

## Abstract

It is well reported that individuals spend up to 90% of their daily time indoors, with between 60% to 90% of this time being spent in the home. Using a cross-sectional study design in a population of 111 healthy adults (mean age: 52.3 ± 9.9 years; 65% women), we investigated the association between exposure to total volatile organic compounds (VOCs) in indoor residential environments and measures of central arterial stiffness, known to be related to cardiovascular risk. Indoor VOC concentrations were measured along with ambulatory measures of pulse pressure (cPP), augmentation index (cAIx) and cAIx normalized for heart rate (cAIx_75_), over a continuous 24-h period. Pulse wave velocity (cfPWV) was determined during clinical assessment. Multiple regression analysis was performed to examine the relationship between measures of arterial stiffness and VOCs after adjusting for covariates. Higher 24-h, daytime and night-time cAIx was associated with an interquartile range increase in VOCs. Similar effects were shown with cAIx_75_. No significant effects were observed between exposure to VOCs and cPP or cfPWV. After stratifying for sex and age (≤50 years; >50 years), effect estimates were observed to be greater and significant for 24-h and daytime cAIx in men, when compared to women. No significant effect differences were seen between age groups with any measure of arterial stiffness. In this study, we demonstrated that residential indoor VOCs exposure was adversely associated with some measures of central arterial stiffness, and effects were different between men and women. Although mechanistic pathways remain unclear, these findings provide a possible link between domestic VOCs exposure and unfavourable impacts on individual-level cardiovascular disease risk.

## 1. Introduction

Exposure to environmental factors such as air pollution have previously been suggested to amplify the effect of traditional risk factors, such as blood pressure and smoking [1,2], on cardiovascular risk [3,4].

Volatile organic compounds (VOCs) comprise a large, diverse and common group of chemical pollutants that are present in both outdoor (ambient) and indoor air. Commonly occurring VOC species include formaldehyde (and other aldehydes), benzene, toluene, xylene and polycyclic aromatic hydrocarbons.

In ambient air, the most significant sources are from vehicle exhausts, while major indoor sources of VOCs include building materials and finishes (primarily flame retardants) such as paints and floor coverings made of, or containing, flexible plastics such as vinyl, engineered wood products, cleaning products and fragranced personal hygiene products [5,6]. Although VOCs detected in indoor environments are usually present at low concentrations, they have been associated with a range of adverse health effects including sensory and skin irritation, headaches, nausea, respiratory illness and cancer [4,5,6,7].

More recently, positive associations between ambient exposure to VOCs and daily emergency hospital admissions for cardiovascular disease (CVD) [4] and heart failure [1] have been reported. 

Among markers of arterial disease, central (aortic) arterial stiffness is well reported as an established marker of subclinical CVD [8]. Whilst aortic wall stiffness increases with advancing age, particularly in the presence of CVD risk factors such as elevated blood glucose and obesity [9,10,11,12], increases have also been observed in relatively healthy individuals with a low burden of traditional vascular disease risk factors, suggesting a background effect of aging per se or possibly reflecting exposure to unknown or nontraditional risk factors [13] such as air pollution.

Studies of ambient [3,14] and indoor air [15] have provided varying evidence that exposure to particulate and gaseous components of air pollution is associated with impaired arterial stiffness. However, none have reported on exposures to VOCs in residential homes where individuals spend greater than two-thirds of their daily time [16,17,18] and where sources of VOCs are numerous and abundant. 

The aim of this cross-sectional study was to investigate associations between indoor residential exposure to VOCs and subclinical measures and correlates of central arterial stiffness in a population of healthy, middle-aged men and women living in Perth, Western Australia. This study did not attempt to determine air exchange rates (ventilation) or characterize emission sources of indoor VOCs.

## 2. Materials and Methods

The population for this study comprised 111 middle-aged men and women, and data were collected in a two-stage process defined as the “home stage” and “clinic stage”. 

This study was approved by the Curtin University, Human Research Ethics Committee, and all participants provided written informed consent.

Detailed methods for this study protocol have been published previously [19].

### 2.1. Inclusion and Exclusion Criteria

Participant inclusion criteria were healthy nonsmoking adults living in nonsmoking households (by self-report), aged 35–69 years, and willing to participate in both stages of the study. Subjects were excluded if they reported history of cardiovascular morbidity or CVD, had medically diagnosed diabetes, used antihypertensive, antidiabetic or lipid-lowering medications or were unable to provide written consent.

### 2.2. Home Stage—24-h Central Ambulatory Monitoring and VOC Measurements

During the home stage, central ambulatory measures of arterial stiffness and in-home VOC concentrations were measured simultaneously over a continuous 24-h period. Both data were recorded at 30-min intervals.

Central ambulatory measures and correlates of arterial stiffness were recorded by a portable monitoring device (Oscar 2, Sun Tech Medical Inc., Morrisville, NC, USA). Measurements obtained included systolic and diastolic blood pressure (BP), augmentation index (cAIx), cAIx normalised for heart rate (cAIx_75_) and pulse pressure (cPP). Twenty-four-hour measurements were calculated as the mean of all readings throughout the 24-h period. Awake and asleep periods were determined from time–activity diaries maintained by participants for the monitoring period using the same method described in other studies [20,21]. Editing of the 24-h measurements was undertaken to reflect self-reported awake and asleep times [21], and measurements were deemed as valid following standard protocols described by Parati et al. [21] and O’Brien et al. [20].

Indoor VOC concentrations were measured at each participant’s residence using scientifically validated instrumentation (Gray Wolf AdvancedSense Pro. Gray Wolf Sensing Solutions, Shelton, CT, USA) [22]. The Gray Wolf AdvancedSense Pro measures VOC concentrations using photoionization detectors (PID) within the range of 5 parts per billion (ppb) to 20,000 ppb, with a resolution of 1 ppb and limit of detection of <5 ppb. This PID is calibrated to isobutylene and measures VOCs to 10.6 eV. It does not respond to VOCs with ionization potentials >10.6 eV, such as ethane, methane or formaldehyde.

Data with respect to health status and domestic indoor environment were collected by questionnaires common to previous similar studies [7,23].

### 2.3. Clinic Stage—Pulse Wave Velocity and Anthropometric Measurements

During the clinic stage, participants attended a clinic-based health assessment that included a structured interview to collect demographic, socioeconomic and lifestyle information along with a current health profile. Anthropometric measures included height, weight and waist and hip circumference. Carotid–femoral pulse wave velocity (cfPWV), indicated as a gold standard measure by the American Heart Association scientific statement [24] and the European expert consensus document [25] was determined during pulse wave analysis by examining central arterial pulse waveforms obtained from the right femoral artery and concurrent direct applanation tonometry of the right common carotid artery. Central pressures were measured with the participant in the supine position using the SphygmoCor device (EM3 XCEL, AtCor Medical Pty, West Ryde, Australia). All recordings were performed on the right side of the body, and transit distances were assessed by body surface measurements from the suprasternal notch to each pulse recording site. Carotid–femoral PWV was determined by examining central arterial waveforms obtained from the common carotid and femoral artery, and the time delay measured between the feet of the two waveforms. The distance covered by the waves was established as 80% of the distance between the two recording sites. All data were collected directly onto a laptop computer and processed with approved waveform analysis using a previously validated method [26].

### 2.4. Statistical Analysis

We compared the difference in cAIx, cAIx_75_, cPP and cfPWV when exposed to indoor residential VOCs in a healthy middle-aged population. Similar to other studies, further subgroup analyses were performed to examine effect differences stratified by sex (male and female) and age (≤50 years and >50 years) [27].

Participant data describing demographic characteristics, physical measurements and clinical characteristics have been stratified by sex and are presented as the mean with standard deviation (SD) for continuous variables and counts with percentages for categorical variables. Differences in susceptible subpopulations were tested using independent samples *t*-tests (male vs. female; ≤50 years vs. >50 years).

Descriptive data were produced to describe mean 24-h concentrations for VOCs and measurements of ancillary variables including temperature and relative humidity. Bivariate association between measures of arterial stiffness (cAIx, cAIx_75_, cPP, cfPWV) and 24-h VOC concentrations, temperature, and relative humidity (RH) were assessed by using the Pearson’s correlation coefficient, *r*.

Multiple regression analysis using the general linear model univariate procedure was performed to investigate the association between each outcome variable and VOC adjusting for confounders or covariates, separately. A basic model (Model 1) was built for all four outcomes without adjusting for any covariates. Age (continuous), sex (nominal; male, female), BMI (continuous), waist–hip ratio (continuous) and SES (ordinal; low, medium, high) were included in Model 2 as a priori CV risk factors [2,15,27,28]. Mean 24-h temperature (continuous), identified as a further theoretically important confounder in the literature, was included in the final model [15,27,28].

Other covariates identified as contributing to the causal mechanisms of vascular damage, such as smoking (tobacco or e-cigarette), the presence of medically diagnosed hypertension (and subsequent use of antihypertensive medications), diagnosed dyslipidaemia (and subsequent use of lipid-modifying medications), historical CV events and/or a medically diagnosed prediabetic or diabetic profile, were eliminated by the inclusion and exclusion criteria of the study. 

To evaluate the influence of residual confounding effects on the final results, relative humidity, waist circumference, ethnicity and alcohol consumption were included in further regression models to explore their influence on estimates. Effect estimates were not altered significantly with their inclusion; therefore, they were excluded from the final model. We then explored for potential effect modification by sex and age (Supplementary material, Tables S1 and S2). 

Effect estimates are presented as the mean change (β) in the outcome variable corresponding to a one interquartile range (IQR) increase in indoor VOC concentrations, along with its 95% confidence interval (CI). A *p*-value ≤ 0.05 was considered to indicate statistical significance. 

All statistical analyses were conducted using IBM SPSS version 26.0 software (IBM Corp., Armonk, NY, USA).

## 3. Results

Of the 111 self-reported healthy middle-aged participants, 65% were women. The mean age of the study population was 52.3 years (SD = 9.9), and most participants lived in areas of higher socioeconomic advantage (79%) (Table 1). 

Women were observed to have significantly higher cAIx and cAIx_75_ when compared to men; however, this was inversed for cPP and cfPWV. No significant difference was observed between the under 50 years and over 50 years age groups in cAIx or cAIx_75_, although a significant difference was shown between under and over 50 years females, in cPP measured over 24 h and during the daytime.

Men over the age of 50 years recorded the highest cfPWV, although there was a significant difference in cfPWV between participants aged under 50 and those aged over 50, in both sexes. However, no significant difference was observed between the sexes (Table 2).

### 3.1. Air Quality and Residential Characteristics

Indoor VOC concentrations along with ancillary variables including temperature and relative humidity were measured over a continuous 24-h period and are presented in Table 3. Correlation coefficients showed no relationship between VOC with any of the measured covariates including 24-h indoor temperature or relative humidity. 

The homes sampled in this study, typically, were stand-alone single-family dwellings (*n* = 96; 86.5%) (compared to a group dwelling defined as having a common wall with a neighboring property), with ≥3 or more occupants (*n* = 66; 59.4%). Eighty-three homes were aged 10 years or greater (74.8%), and 60 (54.1%) had a garage attached to the main part of the home by an adjoining door. Equal numbers of households reported to be located ≤300 m or ≥300 m from a main roadway (*n* = 53; 47.7%). Most houses used a combination of gas (stovetop) and electric (oven) appliances when cooking (*n* = 63; 56.8%), and always or regularly used an extractor fan (*n* = 97; 87.4%). None of the households included a smoker or allowed smoking within the home. 

A summary of the characteristics of the dwellings sampled is presented in Table 4.

### 3.2. Associations with Subclinical Measures of Arterial Stiffness

In Model 1 (unadjusted), no clear associations were observed between domestic indoor VOCs exposure and measures of arterial stiffness (Table 5). In Model 2, controlling for age, sex, BMI, waist-hip ratio and SES, an IQR (124.7 ppb) increase in residential VOC concentration was associated with a 1.04%, 1.23% and 0.91% increase in cAIx measured over 24-h (95% CI: 0.25, 1.84; *p* = 0.011), during the daytime (95% CI: 0.32, 2.14; *p* = 0.009) and during the night-time (95% CI: 0.08, 1.75; *p* = 0.033), respectively.

Similar associations were also observed for cAIx_75_ measured for 24 h (0.90%; 95% CI: 0.10, 1.70; *p* = 0.028), daytime (1.01%; 95% CI: 0.14, 1.89; *p* = 0.023) and night-time (0.87%; 95% CI: −0.05, 1.79; *p* = 0.064). 

In Model 3, with further adjustment for 24-h mean indoor temperature, the relationship was significantly strengthened for 24-h (1.09%; 95% CI: 0.29, 1.90; *p* = 0.008; cAIx_75_: 0.93%; 95% CI: 0.12, 1.74; *p* = 0.025) and daytime (cAIx: 1.33%; 95% CI: 0.42, 2.24; *p* = 0.005; cAIx_75_: 1.11%; 95% CI: 0.24, 1.98; *p* = 0.013) measurements. 

A summary of these results are presented in Table 5.

**Figure 1:**
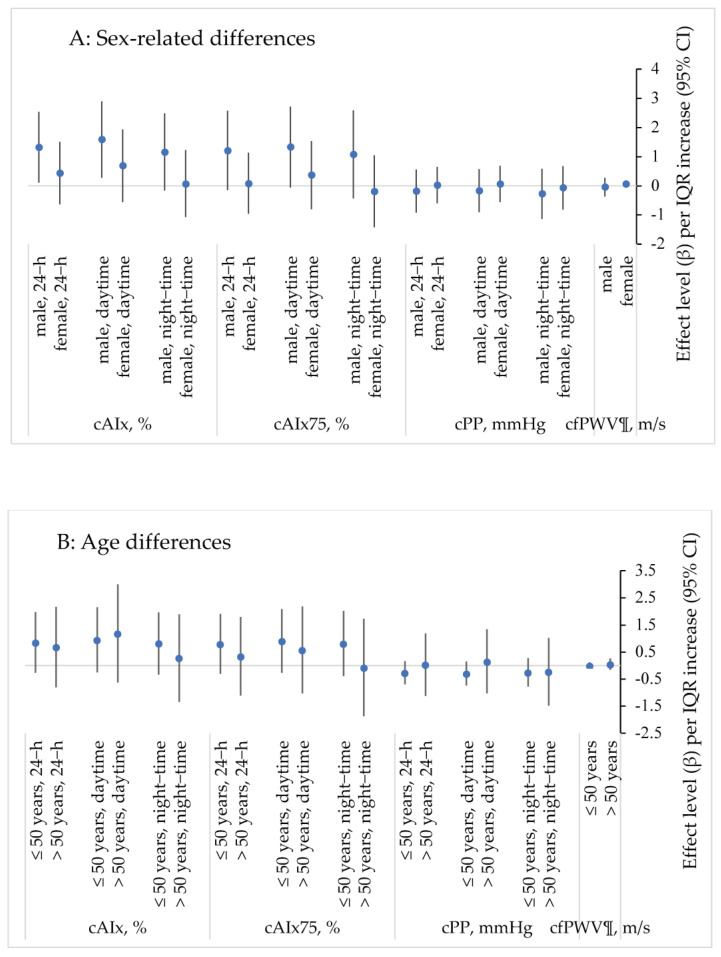
Association between VOCs and measures of arterial stiffness stratified by sex (**A**) and age (**B**). Multiple linear regression; adjusted for age, gender, BMI, waist–hip ratio, socioeconomic status (low, medium, high) and 24-h mean temperature; data are presented as mean change (β) and 95% confidence interval (CI) for one interquartile range (IQR) increase in indoor VOCs; cAIx—central (aortic) augmentation index; cAIx75—central (aortic) augmentation index normalised for heart rate; cPP—central (aortic) pulse pressure; cfPWV—carotid–femoral pulse wave velocity; **¶**—this measurement was obtained during the clinical pulse wave analysis assessment (rather than as an ambulatory measure).

After refitting the same final model for sex and age differences, effect estimates were observed to be greater and significant for 24-h and daytime cAIx and cAIx_75_ in men when compared to women (Figure 1A; Supplementary Material, Table S1). No significant differences in effect were found between the under 50 age group when compared to the over 50 age group (Figure 1B; Supplementary Material, Table S2).

No relationship was established between domestic VOCs exposure and cPP or cfPWV (Table 5; Supplementary Material, Tables S1 and S2).

## 4. Discussion

In this study, we examined the relationship between domestic indoor exposure to total VOCs and subclinical measures of central arterial stiffness in a healthy middle-aged population of men and women, residing in Perth, Western Australia. To our knowledge, this is the first study to investigate the association between exposure to residential indoor VOCs and measures and correlates of arterial stiffness; although, in a South African study of 77 healthy female adults, Everson and colleagues were able to demonstrate associations between low exposure levels of some VOCs (benzene, toluene ethyl-benzene and m + *p*- and o-xylenes (BTEX)) and several cardiovascular haemodynamic parameters, including systolic and diastolic BP [2]. 

In the current study, we found that increasing exposure to domestic indoor VOCs was associated with an elevated augmentation index (and cAIx_75_), and that effects were greater in men when compared to women. However, no differences were observed for the association of VOCs with any measure of arterial stiffness among those individuals who were aged below or above 50. Ran et al. also reported similar sex-different and age-related results in a study aiming to estimate the effects of ambient VOCs on heart failure emergency hospitalisations in a population-based cohort (*n* = 54,003) located in Hong Kong [1]. 

Arterial stiffness, recognized as an established marker of vascular health, provides independent predictive information on the risk of adverse CV outcomes beyond that of established measures such as peripheral BP. Measures and correlates of arterial stiffness such as cAIx, cAIx_75_, cPP and cfPWV are indicators of overall CV performance, with higher values representing increased risk for adverse CV outcomes [29,30]. Whilst studies of ambient air have provided varying evidence that exposure to particulate air pollution and some gaseous pollutants is associated with impaired arterial stiffness [3,14], to our knowledge, no previous studies have assessed how exposure to residential indoor VOCs impacts subclinical haemodynamic measures of arterial stiffness such as cAIx, cAIx_75_, cPP or cfPWV. 

In the current study, the greatest and most consistent significant effects were observed between VOCs and cAIx and cAIx_75_, with increases of >1% observed in adjusted models for 24-h and daytime cAIx and cAIx_75_ in the total population. Although the nocturnal effect was slightly less, significant and unfavourable increases in cAIx (marginal for cAIx_75_) were also observed. 

Whilst we were unable to identify any previous studies that have reported on the relationship between VOCs exposure and any measure of arterial stiffness, potential explanations for our findings could be found in the literature. 

Increased arterial stiffness is associated with vascular damage that can be either structural or functional in nature, and traditional CVD risk factors such as hypertension and dyslipidaemia are known to contribute to both structural and functional vascular damage [31,32]. It is also well-established that the average man is at greater CV risk than his female counterpart is, with most theories proposing that female sex hormones may be responsible for better arterial distensibility among women [33,34].

Arterial stiffness is also the principal cause of CVD with age [11,12,31], and advancing age is associated with hypertension and the risk of CV events [27]. These outcomes are better described by a process of underlying *structural* change [11,12,31].

Systemic inflammatory responses have also been suggested as the primary mechanism by which inhaled air pollution induces adverse CV response [14,35]. Increased inflammatory markers as might be seen in inflammatory conditions such as rheumatoid arthritis have been linked to adverse vascular changes, including atherosclerosis, hypertension and increased central arterial stiffness [27,36,37], causing functional stiffening of the arteries [31]. 

Because exposure to air pollution has been linked to adverse alterations of blood biomarker levels that stimulate inflammation and endothelial dysfunction [27,38] (these conditions are both associated with functional arterial stiffening) [32,39], it is conceivable that inflammation might be responsible for the stiffening of large arteries after exposure to air pollution (including VOCs), even at acute, low-level and transitory exposure, and might be related to a functional arterial stiffening response [32]. Additionally, evidence also exists to suggest that exposure to air pollution is associated with acute arterial vasoconstriction [38,40], which may lead us to conclude that the increases in cAIx seen in our healthy population could be the result of air pollution-mediated vasoconstriction at microcirculation level. This can result in the early arrival of the return of pulse wave reflection from the periphery [11], reflecting functional rather than structural changes in arterial stiffness. This is consistent with the view of Adamopoulos et al. [3] and Zanoli et al. [32].

In this current study, we observed associations between VOCs exposure and cAIx; however, not with cfPWV. Potential explanation for this finding is provided by Kelly and colleagues [41], who propose that alterations in cfPWV and cAIx can occur independently, depending on which section of the arterial tree is most influenced by the exposure. The mechanisms of stiffening differ according to the region of the arterial tree because the properties of the arterial wall vary along the longitudinal axis of the arterial tree (e.g., elastic arteries are dominated by elastin fibres and muscular arteries are dominated by collagen and smooth muscle cells) [11,24,31].

That said, and in the context of the present study, it is conceivable that short-term recent exposures to all or selected VOCs might have altered endothelial function in conduit and elastic arteries, with the intermediate but potentially transient effect of increasing arterial stiffness. However, this hypothesis requires further investigation to clarify the relationship and whether, if this theory is correct, reduction of exposure to this group of pollutants or individual VOCs is associated with a reduction in arterial stiffness. 

This study has several strengths above the significant contribution it adds to the currently limited body of evidence related to the impact of residential VOCs exposure on important measures and correlates of arterial stiffness known to be associated with increased CV risk. 

Firstly, this study used directly measured and continuous indoor air quality data combined with widely accepted measures of arterial stiffness and wave reflection. The use of directly measured data potentially reduces the opportunity for introduced bias related to exposure and outcome misclassification, reported as a limitation in other studies that have relied on surrogates and self-reported data [42,43]. We did however observe a higher standard deviation in TVOC concentrations. Although identifying the specific reasons for this was beyond the scope of the current study, it may represent the impact of participant activities and/or household characteristics (such as opening/closing windows, cleaning activities, cooking) which are known to influence domestic VOC concentrations [44,45].

Secondly, this study benefitted from a relatively homogenous random sample of apparently healthy, well-characterised, middle-aged adults living in a geographical area where outdoor air pollution (which contributes to total exposure) concentrations are fairly consistent, and typically below accepted air quality standards. The nature of this study population conceivably aided in the minimisation of effect modifications by other potential factors (such as BMI or socioeconomic status).

However, the study is limited by the cross-sectional design, which does not allow the establishment of a temporal relationship and provides no indication of the evolution of events. The observed impact on measures and correlates of arterial stiffness at one time point may have occurred before the onset of adverse health effects due to VOCs exposure. It is therefore not possible to evaluate the potential for causality in the reported associations. Additionally, it is not known which individual VOCs are responsible for the observed alterations to vascular function seen in this study. Future work should explore potential effects of exposure to individual species of VOCs with arterial stiffness.

This study also reports associations between 24-h averaged VOC data and suboptimal CV health outcomes. It is well reported that there is daily variability in domestic VOC concentrations related to household characteristics and occupant activities [45] and that VOCs may have rapid or immediate impacts upon the human CV system [46]. Whilst time-resolving VOC concentrations with the onset of adverse CV outcomes was beyond the scope of this study, future studies should be undertaken using a different study design to better understand the exposure–response relationship.

## 5. Conclusions

To the best of our knowledge, this is the first study to investigate domestic VOCs exposure with subclinical measures of CV health related to central vascular function. VOCs are ubiquitous in domestic indoor environments, and exposure to VOCs indoors, where individuals spend large portions of their daily time, may have important implications for CV health. The results of this current study suggest that residential indoor exposures to VOCs may potentially and adversely affect some measures of arterial stiffness (cAIx and cAIx_75_). Importantly, these findings were observed in a healthy population of middle-aged adults with greater effects noted in men, when compared to women. Future research should establish whether these effects are amplified in other populations, most notably in individuals at greater risk for CVD, or among those who already may have compromised vasculature (such as individuals with diabetes or hypertension). Arterial stiffness is increasingly recognized as a risk factor for CVD, independent of traditional risk factors, and exposure to harmful pollutants in the indoor environment should not be neglected. Consideration should be given to improving indoor air quality through various means, including the selection of building materials and indoor furnishings, building construction and indoor activities, with an emphasis on lowering the emissions of VOCs to as low as is reasonably achievable. 

## Figures and Tables

**Table 1:** Sociodemographic and physical profile of the study population.

	Men	Women
	Mean ± SD	Mean ± SD
**Demographics**		
Age (years)	50.7 ± 10.7	53.2 ± 9.3
*n* (%)	39 (35.1)	72 (64.9)
SES, *n* (%)		
Low	8 (7.2)	
Medium	15 (13.5)	
High	88 (79.3)	
Do you suffer from any chronic conditions? *n* (%)		
None	83 (74.8)	
Asthma, thyroid etc.	28 (25.2)	
Medications, *n* (%)		
None	49 (44.1)	
Vitamin supplements	27 (24.3)	
Prescription medication ^§^	23 (20.7)	
Combination of vitamins and prescription ^§^	12 (10.8)	
**Physical measurements**		
Height (cm)	178.8 ± 8.6	163.2 ± 6.4
Weight (kg)	82.7 ± 10.8	65.0 ± 10.4
BMI (kg/m^2^)	25.8 ± 2.8	24.4 ± 3.5
Waist measurement (cm)	92.8 ± 8.7	80.0 ± 10.6
Hip–waist ratio	0.92 ± 0.06	0.81 ± 0.06

*n* = 111; SD—standard deviation; SES—socioeconomic status; ^§^—these are medications other than antihypertensive, antidiabetic or lipid-lowering medications, which were part of the exclusion criteria; cm—centimetres; kg—kilograms; BMI—body mass index.

**Table 2:** Clinical profile of the study population.

	Male	Female	*p*-Value
	Mean ± SD	Mean ± SD	
cSBP ^†^, mmHg			
24-h	111.8 ± 10.7	105.9 ± 8.7	0.005
Daytime	118.0 ± 11.6	110.7 ± 8.4	≤0.001
Night-time	101.7 ± 12.3	95.9 ± 10.2	0.011
Cdbp ^†^, mmHg			
24-h	74.5 ± 7.6	68.6 ± 6.7	≤0.001
Daytime	79.7 ± 8.1	73.8 ± 6.7	≤0.001
Night-time	63.4 ± 9.2	58.5 ± 7.6	0.003
HR ^†^, bpm	68.0 ± 8.3	72.0 ± 7.0	0.016
CAIx ^†^, %			
24-h	32.16 ± 9.08	41.63 ± 8.03	≤0.001
Daytime	31.11 ± 10.05	39.77 ± 9.41	≤0.001
Night-time	34.61 ± 9.23	45.34 ± 8.70	≤0.001
cAIx_75_^†^, %			
24-h	28.34 ± 9.70	39.56 ± 7.80	≤0.001
Daytime	29.24 ± 9.79	39.69 ± 8.79	≤0.001
Night-time	26.58 ± 10.72	39.69 ± 9.47	≤0.001
cPP ^†^, mmHg			
24-h	38.34 ± 6.18	37.07 ± 5.14	0.257
Daytime	38.29 ± 6.59	36.94 ± 5.22	0.246
Night-time	38.26 ± 6.29	37.46 ± 5.74	0.502
cfPWV ^‡^, m/s	7.1 ± 1.4	6.9 ± 1.1	0.081

*n* = 111; SD—standard deviation; ^†^—taken from 24-h ambulatory measurements; ^‡^—measured in the supine position during pulse wave analysis; cSBP—central systolic blood pressure; cDBP—central diastolic blood pressure; HR—heart rate; bpm—beats per minute; cAIx—central (aortic) augmentation index; cAIx_75_—central (aortic) augmentation index normalised for heart rate; cPP—central (aortic) pulse pressure; cfPWV—carotid femoral pulse wave velocity; *p*-value—independent samples *t*-test for the difference between male and female.

**Table 3:** 24-Hour mean air quality characteristics of measured households.

	Mean ± SD	Min–Max
Temperature, °C	23.6 ± 3.0	17.0–29.6
RH, %	49.2 ± 8.2	26.6–72.2
VOC, ppb	406.6 ± 272.0	97.6–1888.4

*n* = 111; SD—standard deviation; RH—relative humidity; min—minimum; max—maximum; °C —degrees celcius; VOC—volatile organic compounds, ppb—parts per billion.

**Table 4:** Household characteristics of the study population.

Characteristic	*n* (%)
Type of home	
Stand-alone dwelling	96 (86.5)
Group dwelling	15 (13.5)
Age of the home	
<10 years	26 (23.4)
>10 years	83 (74.8)
Number of occupants	
≤2	43 (38.7)
≥3	66 (59.4)
Garage attached	
Yes	60 (54.1)
No	49 (44.1)
Type of cooking appliances	
Gas	25 (22.5)
Electric	20 (18.0)
Both	63 (56.8)
Use of cooking extractor fan	
Always/usually	97 (87.4)
Never	12 (10.9)
Distance to a major roadway	
≤300 m	53 (47.7)
≥300 m	53 (47.7)
Cleaning frequency	
Several times per week	77 (69.4)
Irregularly	31 (27.9)
Type of floor coverings	
Carpet, linoleum	29 (26.1)
Stone, concrete	30 (27.0)
Wood	50 (45.0)

**Table 5:** Estimated effect (β) of an IQR increase in indoor VOC concentrations on measures of arterial stiffness.

		cAIx, %			cAIx_75_, %			cPP, mmHg			cfPWV ^§^, m/s
		24-h	Daytime	Night-Time	24-h	Daytime	Night-Time	24-h	Daytime	Night-Time	
Model 1	β estimate	0.69	0.88	0.53	0.55	0.64	0.51	−0.15	−0.14	−0.20	−0.01
	95% CI	−0.15, 1.53	−0.04, 1.79	−0.39, 1.45	−0.34, 1.43	−0.27, 1.56	−0.54, 1.56	−0.65, 0.36	−0.66, 0.39	−0.74, 0.35	−0.14, 0.11
	*p*-value	0.107	0.061	0.257	0.227	0.165	0.340	0.562	0.606	0.474	0.823
Model 2	β estimate	1.04	1.23	0.91	0.90	1.01	0.87	−0.17	−0.17	−0.21 L33	0.04
	95% CI	0.25, 1.84	0.32, 2.14	0.08, 1.75	0.10, 1.70	0.14, 1.89	−0.05, 1.79	−0.62, 0.28	−0.63, 0.29	−0.73, 0.31	−0.08, 0.16
	*p*-value	0.011	0.009	0.033	0.028	0.023	0.064	0.455	0.457	0.428	0.560
Model 3	β estimate	1.09	1.33	0.88	0.93	1.11	0.80	−0.17	−0.17	−0.22	0.03
	95% CI	0.29, 1.90	0.42, 2.24	0.03, 1.72	0.12, 1.74	0.24, 1.98	−0.13, 1.73	−0.63, 0.28	−0.64, 0.30	−0.74, 0.31	−0.09, 0.15
	*p*-value	0.008	0.005	0.042	0.025	0.013	0.090	0.454	0.472	0.417	0.593

*n* = 111; multiple linear regression; data are presented as mean change (β) and 95% confidence interval for one IQR increase in indoor VOCs (124.7 ppb); **^§^**: measured in supine position during pulse wave analysis. Model 1: unadjusted; Model 2: adjusted for age, gender, BMI, waist–hip ratio and socioeconomic status (low, medium, high); Model 3: adjusted as per Model 2 + 24-h mean indoor temperature.

## Supplementary Materials

The following are available online at www.mdpi.com/article/10.3390/ijerph19020981/s1, Table S1: Estimated effect (β) of an IQR increase in indoor VOC concentrations on measures of arterial stiffness in a sex-stratified population. Table S2: Estimated effect (β) of an IQR increase in indoor VOC concentrations on measures of arterial stiffness in an age-stratified population.
