# Supplementary material for: The Association between Exposure to Residential Indoor Volatile Organic Compounds and Measures of Central Arterial Stiffness in Healthy Middle-Aged Men and Women
Source: Int J Environ Res Public Health. 2022 Jan 16;19(2):981. doi: 10.3390/ijerph19020981 (PMC8776238; doi:10.3390/ijerph19020981)
Supplement: Supplementary file 1 [file ijerph-19-00981-s001.zip › ijerph-1522548-supplementary.pdf]

Supplementary material

Table S1. Estimated effect (β) of an IQR increase in indoor VOC concentration on measures of arterial stiffness in a sex stratified population.

		**cAIx**			**$cAIx_{75}$**			**cPP**			**cfPWV ¶**
		**24-hour**	**Daytime**	**Nighttime**	**24-hour**	**Daytime**	**Nighttime**	**24-hour**	**Daytime**	**Nighttime**	
**Men** (*n* = 39)		1.32	1.59	1.16	1.21	1.33	1.08	-0.18	-0.17	-0.27	-0.04
	95% CI	0.11, 2.54	0.28, 2.89	-0.16, 2.48	-0.15, 2.57	-0.06, 2.71	-0.43, 2.59	-0.92, 0.56	-0.90, 0.57	-1.13, 0.59	-0.36, 0.28
	p-value	***0.034***	***0.019***	**0.082**	**0.079**	**0.060**	0.155	0.622	0.645	0.525	0.805
**Women** (*n* = 72)		0.44	0.69	0.07	0.08	0.37	-0.19	0.03	0.07	-0.06	0.06
	95% CI	-0.63, 1.51	-0.56, 1.93	-1.07, 1.22	-0.96, 1.13	-0.80, 1.53	-1.42, 1.05	-0.60, 0.65	-0.56, 0,69	-0.81, 0.68	-0.06, 0.18
	p-value	0.410	0.273	0.901	0.872	0.529	0.763	0.931	0.833	0.863	0.318

multiple linear regression; adjusted for age, gender, BMI, waist-hip ratio, socioeconomic status (low, medium, high) and 24-hour mean temperature; data are presented as mean change (β) and 95% confidence interval for one IQR increase in indoor VOC (124.7 ppb); ¶: measured in supine position during pulse wave analysis

Table S2. Estimated effect (β) of an IQR increase in indoor VOC concentration on measures of arterial stiffness in an age stratified population.

		cAIx			$cAIx_{75}$			cPP			cfPWV [¶]
		**24-hour**	**Daytime**	**Nighttime**	**24-hour**	**Daytime**	**Nighttime**	**24-hour**	**Daytime**	**Nighttime**	
**≤ 50 years**		0.82	0.92	0.79	0.77	0.88	0.78	-0.30	-0.33	-0.28	-0.03
	95% CI	-0.33, 1.98	-0.32, 2.16	-0.40, 1.97	-0.38, 1.91	-0.33, 2.09	-0.46, 2.02	-0.76, 0.17	-0.80, 0.15	-0.85, 0.28	-0.18, 0.12
	p-value	0.156	0.140	0.186	0.183	0.149	0.211	0.200	0.177	0.315	0.683
**> 50 years**		0.65	1.16	0.25	0.31	0.55	-0.10	0.00	0.12	-0.26	0.02
	95% CI	-0.87, 2.17	-0.69, 3.00	-1.41, 1.90	-1.18, 1.80	-1.09, 2.19	-1.94, 1.73	-1.19, 1.19	-1.09, 1.34	-1.55, 1.03	-0.22, 0.26
	p-value	0.394	0.214	0.767	0.682	0.502	0.910	0.995	0.838	0.684	0.860

multiple linear regression; adjusted for age, gender, BMI, waist-hip ratio, socioeconomic status (low, medium, high) and 24-hour mean temperature data are presented as mean change (β) and 95% confidence interval for one IQR increase in indoor VOC (124.7 ppb); [¶]: measured in supine position during pulse wave analysis
